# Treatment of Granulomas in Patients With Ataxia Telangiectasia

**DOI:** 10.3389/fimmu.2018.02000

**Published:** 2018-09-18

**Authors:** Sandra Woelke, Eva Valesky, Shahrzad Bakhtiar, Helena Pommerening, L. M. Pfeffermann, Ralf Schubert, Stefan Zielen

**Affiliations:** ^1^Division of Allergology, Pulmonology and Cystic Fibrosis, Department for Children and Adolescents, Goethe University, Frankfurt, Germany; ^2^Department of Dermatology, Venereology and Allergology, Goethe University, Frankfurt, Germany; ^3^Division for Stem Cell Transplantation and Immunology, Department for Children and Adolescents, Goethe University, Frankfurt, Germany

**Keywords:** ataxia telangiectasia, granulomas, granulomatous inflammation, TNF inhibitors, primary immunodeficiency

## Abstract

**Background:** Ataxia telangiectasia (A-T) is a devastating multi-system disorder characterized by progressive cerebellar ataxia, growth retardation, immunodeficiency, chronic pulmonary disease and chromosomal instability. Cutaneous granulomas are a known phenomenon in A-T but extra-dermal manifestation of granulomas at bone and synovia has not been reported so far. The clinical presentation, immunological findings, the long-term course and treatment options of eight patients with severe granulomas will be reported.

**Methods:** From our cohort of 44 classical A-T patients, eight patients aged 2–11 years (18.2%) presented with granulomas. Immunological features of patients with and without granulomas were compared. Five patients suffered from cutaneous manifestation, in two patients we detected a bone and in one a joint involvement. Patients with significant extra-dermal involvement as well as one patient with massive skin manifestation were treated with TNF inhibitors. The patient with granulomas at his finger joint and elbow was treated with hematopoietic stem cell transplantation (HSCT).

**Results:** Interestingly, seven of eight patients with granulomas were total IgA deficient, but there were no differences in IgG and IgM levels. All lymphocytes subsets were equally distributed except patients with granuloma had significantly lower naïve CD8 cells. In patients without treatment, four of eight showed a slow but significant enlargement of the granuloma. Treatment success with TNF inhibitors was variable. In one patient, treatment with TNF inhibitors led to a total remission for 3 years up to now. In two patients, treatment with TNF inhibitors led to a partial regression of granulomas. Treatment interruptions caused deterioration again.

**Conclusions:** Granulomas in A-T progress slowly over years and can lead to significant morbidity.Treatment with TNF inhibitors was safe and in part successful in our patients. Interestingly HSCT leads to complete remission, and indicates that aberrant immune function is responsible for granulomas in A-T patients. **What This Study Adds to the Field:** Granulomas in A-T progress slowly over years and can lead to significant morbidity. Treatment with TNF inhibitors was safe and in part successful in our patients.

**AT A GLANCE COMMENTARY**:

**Scientific knowledge on the subject:** Little is known about the clinical presentation, course and treatment of granulomas in ataxia telangiectasia (A-T). In addition, this is the first report of extra-dermal manifestation of granulomas at bone and synovia in patients with A-T.

**What This Study Adds to the Field:** Granulomas in A-T progress slowly over years and can lead to significant morbidity. Treatment with TNF inhibitors was safe and in part successful in our patients.

## Introduction

Ataxia telangiectasia (A-T) is a rare autosomal recessive multisystem disorder characterized by progressive cerebellar ataxia, neurodegeneration, immunodeficiency and cancer predisposition (1). Additional clinical features of this disease are oculocutaneous telangiectasias, frequent bronchopulmonary infections, growth retardation, gonadal dysgenesis, premature aging and hypersensitivity to ionizing radiation (2–5). The ataxia telangiectasia mutated (*ATM*) gene is located on chromosome 11q22-23 and encodes a 370-kDa protein that belongs to the phosphatidylinositol 3-kinase (PI3-kinase) family of signal transduction proteins (6, 7). ATM is a serine/threonine protein kinase that mediates activation of multiple signaling pathways following the induction of DNA double-strand breaks and oxidative stress (8). Death in affected patients is usually due to lymphoreticular malignancy or chronic lung failure (9). At present, there is no therapy available to cure or prevent the progress of A-T, and its medical management is directed at the associated problems (10).

Besides the above-named symptoms, a multitude of skin manifestations such as dermal telangiectasias, café-au-lait macules or prematurely hair graying as well as cutaneous granulomas can be seen in A-T (11–13). Granulomas have been described by several authors in chromosomal breakage syndromes such as A-T or Nijmegen Breakage Syndrome (NBS) as well as in other primary immunodeficiencies (PID) like common variable immunodeficiency (CVID), severe combined immunodeficiency or hyper-IgM-syndrome (14―17). While in healthy persons, granulomas usually occur in consequence of infections or as foreign body reactions, an obvious trigger mechanism for the origin of granulomas in A-T is often missing (18–20). In A-T granulomas have been described in the so called “hyper IgM phenotype” characterized by decreased IgG and IgA levels with simultaneously normal to elevated IgM levels (15). Chiam et al. (15) proposed that not only in A-T but also in other combined immunodeficiencies such as RAG deficiency, NBS or CVID an imbalance between macrophages in the skin activated by natural killer cells and/or T cells and an insufficient counteracting downregulation of this activation via interleukin 10 promotes the development of granulomas.

Due to the sporadic occurrence in an anyway small cohort of patients there is no generally recommended therapy so far. Antibiotic treatment remained without any resounding success. The treatment with topical steroids was successful in a single case report (21). Only the use of TNF inhibitors was found to be an effective therapy in several reports (22, 23).

Cutaneous granulomas are a known phenomenon in A-T but extra-dermal manifestations of granulomas at bone and synovia have not been reported so far. The clinical presentation, immunological findings, the long-term course and treatment options of eight patients with severe granulomatous inflammation will be reported.

## Materials and methods

We examined 44 classical A-T patients in our inpatient and outpatient clinic between December 2010 and December 2016. All patients were clinically and/or genetically diagnosed with A-T. In case of clinically suspected granulomatous inflammation biopsies were taken and Hematoxylin and eosin-, Periodic acid-Schiff and Grocott staining was done with all biopsies. Furthermore, Ziehl-Neelsen reaction and Gram-staining for exclusion of mycobacterial or other bacterial structures were performed. In case of a suspected extra-dermal manifestation, MRI scan of the suspicious area was done.

In all patients, serum levels of IgG with subclasses, IgA and IgM were determined by nephelometry. Lymphocyte phenotyping was performed using whole blood samples of all patients containing EDTA, which were stained with the following monoclonal antibodies directly labeled either with fluorescein-isothiocyanate, phycoerythrin or PerCP as described (24): UCHT-1 (CD3), B9·11 (CD8), J4·119 (CD19), ALB11 (CD45RA), UCHL-1 (CD45RO) from Coulter-Immunotech (Marseille, France), from Becton Dickinson (San Jose, CA, USA). After staining, erythrocytes were lysed (FACS-lysing solution, Becton Dickinson), washed with PBS/0·1% Na azide and fixed with 1% paraformaldehyde. Measurements were performed on a FC500 flow cytometers (Beckman Coulter). Precise gating was done with computer software (CXP, Beckman Coulter).

## Results

### Clinical presentation

**Figure 1:**
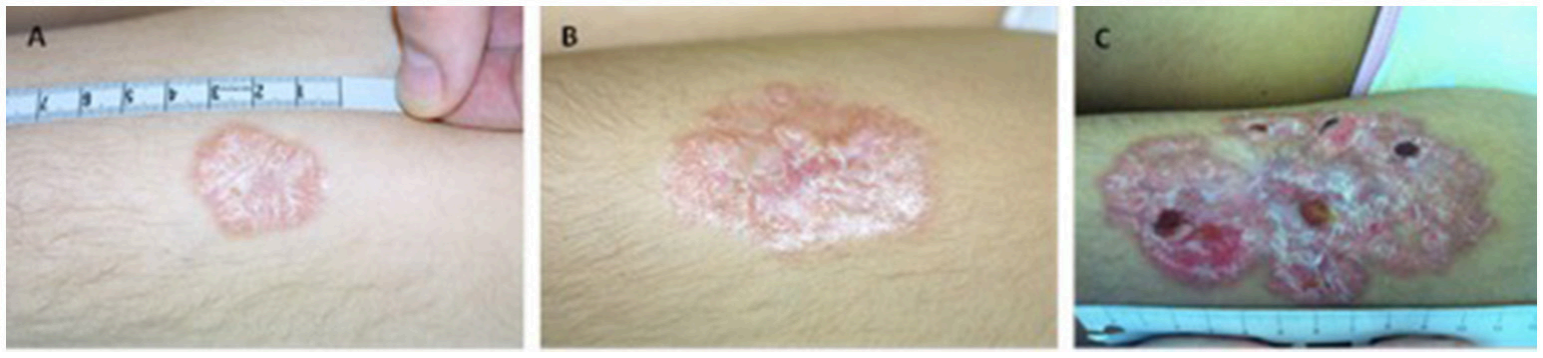
Granulomas in A–T do slowly progress over years in patient 4. **(A)** at manifestation **(B)** after 3 years and **(C)** after 7 years.

From our cohort eight patients (18.2%) presented with granuloma (Table 1). Patients were aged between 2 and 11 years at diagnosis of granulomas. Treatment and out-come of patients were followed median 8 range 2–12 years. Five patients suffered from cutaneous granulomas of varying degree (patients 1–5) (Figures S1, S2, S3). In our patients, the granulomatous skin changes were found to be well-defined erythematous indurated papules and plaques. As shown in Figure 1 (patient 4) granuloma was small in the beginning (less < 5 cm) and progressed after 7 years to > 10 cm with scaly atrophy and ulcerations. Progression of skin granuloma was seen in all patients without treatment during follow-up.

**Table 1:** Patients characteristics with granulomas.

**Patient**	**ATM mutation**	**Age at manifestation (range in years)**	**Granuloma manifestation/localization**	**Follow-up (years)**	**Treatment /duration (years)**	**Outcome**
1	c.2921 + 1G > A c.3320T > A	0–5	Skin/knee	7	None	Progress
2	c.5846A > G c.4673C > T	0–5	Skin/multiple localizations	12	None	Progress
3	c.8781_8786 + 2del Homozygous	0–5	Skin/multiple localizations	2	Ig (since age 2)	Progress
4	Unknown	6–10	Skin/thigh	10	Ig (since age 1)	Progress
5	Unknown	6–10	Skin/lower leg	12	Adalimumab (1.7, ongoing) Ig (since age 8)	Partial regression
6	c.2413C > T c.6095G > A	0–5	Synovia/knee	6	Infliximab (1), Ig (since age 4)	Remission
7	c.5932G > T 2nd mutation missing	11–15	Bone/tibia	8	Infliximab (2.8) Adalimumab (3.8, ongoing) Ig (since age 11)	Partial regression
8	c.480_484del TCAGC c.3206delC	0–5	Joint/finger Skin/elbow	6	BMT (at age of 5)	Remission

*Ig, Immunoglobulin substitution*.

**Figure 2:**
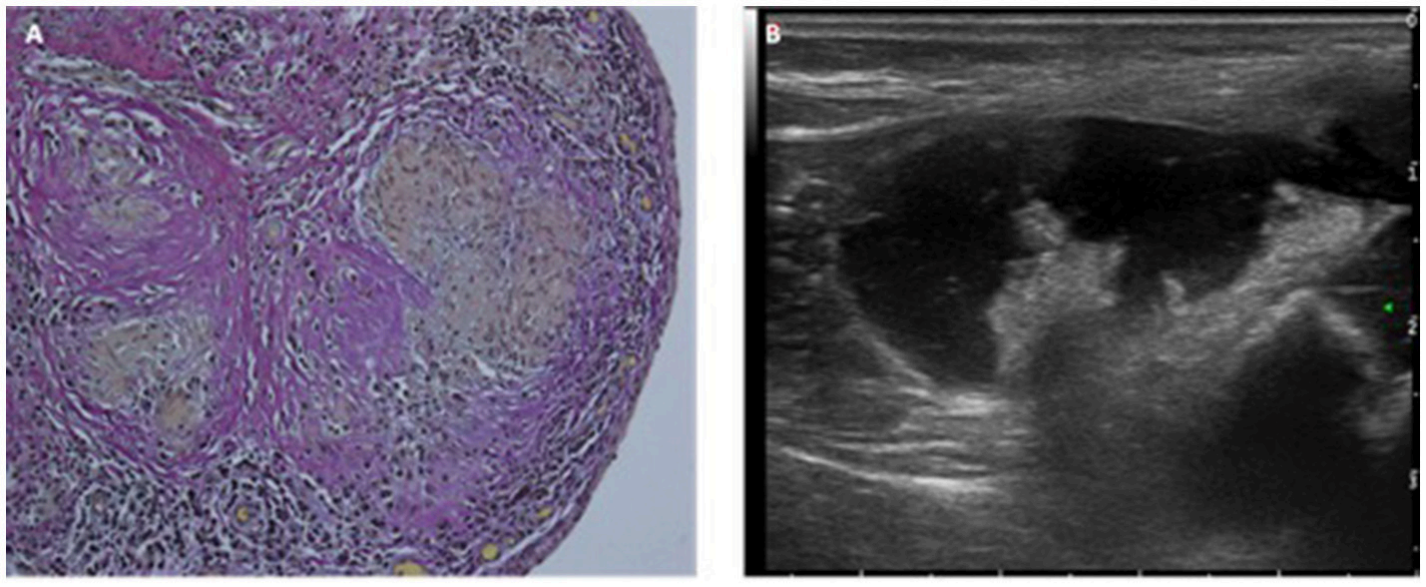
**(A)** Histopathology and **(B)** ultrasound of granulomas at the synovia of patient 6.

Three patients suffered additionally to granulomatous skin changes from extra-dermal manifestation with granulomas of bone, ankle and synovia. The clinical picture of patients with extra-dermal manifestation was as follows:

Patient 6 presented at the age of 4 years with fever, sudden acute painful swelling of his knee consistent with an osteomyelitis. In the ultrasonic examination, an effusion and granulomatous formations in the joint could be seen (Figure 2B). Initially, elevated inflammatory parameters declined after antibiotic treatment but swelling persisted over months. Histopathology of skin and synovia biopsies showed granulomatous inflammation (Figure 2A).

**Figure 3:**
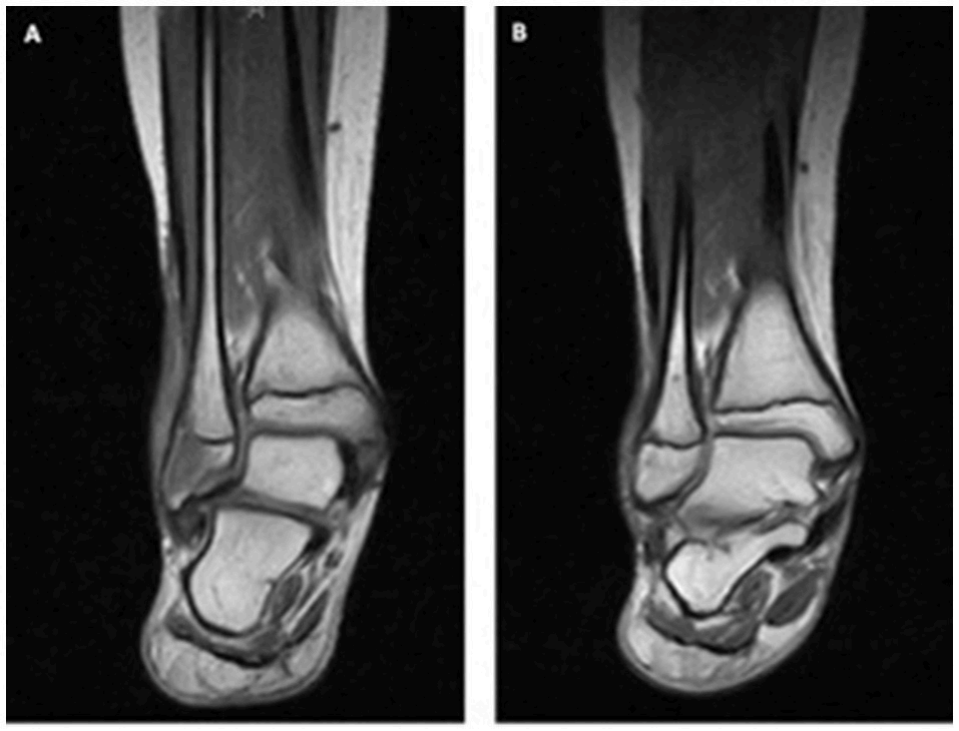
Granulomas in the bone of patient 7. MRI **(A)** at the beginning and **(B)** after 1 year of TNF inhibitor treatment.

Patient 7 presented at the age of 11 years with impaired wound healing of a small skin lesion at the right lower leg. This wound was initially 0.5 cm in size. The patient's skin lesions progressively enlarged with little response to topical treatment and in one of the cultures *Pseudomonas* was detected. However, local wound therapy and parental antibiotic treatment against *Pseudomonas* did not improve healing. The size of the lesion increased to 2 × 3 cm. Six months' later parents noticed a swelling of the outer malleolus at the right leg. Laboratory work up showed no inflammatory response (sedimentation rate 10 mm/h, CRP < 0.5 mg/dL). MRI scan (Figure 3) revealed signs of acute osteomyelitis and a biopsy was taken from the involved bones and from the wound. A 7-day course of parenteral treatment with meropenem and fosfomycin was started. Cultures and PCRs were negative except for a slow growing of *Streptococcus constellatus*. Streptococcus constellatus is a viridans Streptococcus and is associated with abscesses in children (24). This pathogen was susceptible to treatment with meropenem and fosfomycin The histological finding of the skin lesion showed ulcerations and small granulomas with fibrinous necrosis. In the bone tissue, granulomatous inflammation was described.

Patient 8 developed at the age of 4 years a swelling of the proximal interphalangeal joint of his middle finger and small skin lesions at his elbow without further signs of inflammation (Figure S4). Tissue samples of both lesions were taken showing non-infectious granulomas. At the age of 5 years the patient was treated with stem cell transplantation (HSCT).

### Treatment and outcome

In five patients with skin manifestations (patients 2, 3, 4, 5, and 7) treatment with various ointments containing tacrolimus and/or highly potent corticosteroids were not successful. As shown in Table 1 all skin granulomas were progressive. Due to significant morbidity both patients with bone/synovia involvement (patients 6 and 7) as well as one patient with massive skin manifestation (patient 5) were treated with TNF inhibitors. Before treatment was started silent tuberculosis infection was excluded by interferon-γ-release-test. In addition, all patients received regular immunoglobulin substitution if such a treatment was not routine practice. In our cohort of 44 A-T patients only 14 (31.8%) were treated by immunoglobulin substitution, This percentage is line with a recent survey, since many centers do use immunoglobulin substitution only in A-T patients with frequent infections (25) Patient 6 was treated with infliximab (Remicade^®;^) at a dose of 5mg/kg initially and after 14 days, followed by one injection 4 weeks later. As already after one dose of infliximab the effusion in the joint was significantly regressive the interval between infusions was prolonged to 6 weeks. Ultrasonic examinations were performed before each infliximab infusion as well as a MRI scan of the joint before treatment start and after 12 months of treatment. The MRI scan after 12 months showed no granulomas in the joint and only a residual thickening of the synovial membrane in the sense of a restitution. So, treatment with infliximab was stopped and 4 years later the patient is still in a complete remission.

In patient 7, treatment with infliximab was started after cultures and PCRs were negative for infectious pathogens at a dose of 5mg/kg in a 2-week interval for the first 4 months and subsequently continued 4-weekly with regression of skin and bone granulomas during the first 15 months of treatment. A trial discontinuation led to a rapid deterioration with new cutaneous granulomatous formations within 2 months, therefore treatment with infliximab was continued again for another period of 17 months. At the end of this period the effect of infliximab seemed to diminish and cutaneous granulomas were progressive again. Although anti TNF inhibitor antibodies could not be detected, treatment was replaced by adalimumab (Humira®) subcutaneously (at a dose of 40 mg every 2 weeks). Under this treatment granulomatous inflammation was initially regressive again and in the further course stable for a period of 27 months. A second trial discontinuation again led to deterioration of cutaneous granulomas with ulcerations. Therefore, treatment with adalimumab was restarted. The patient has been followed up for 8 years since manifestation of granulomas up to now. In this patient, treatment with TNF inhibitors resulted in a significant regression of bone and skin granulomas. All treatment interruptions caused deterioration again.

**Figure 4:**
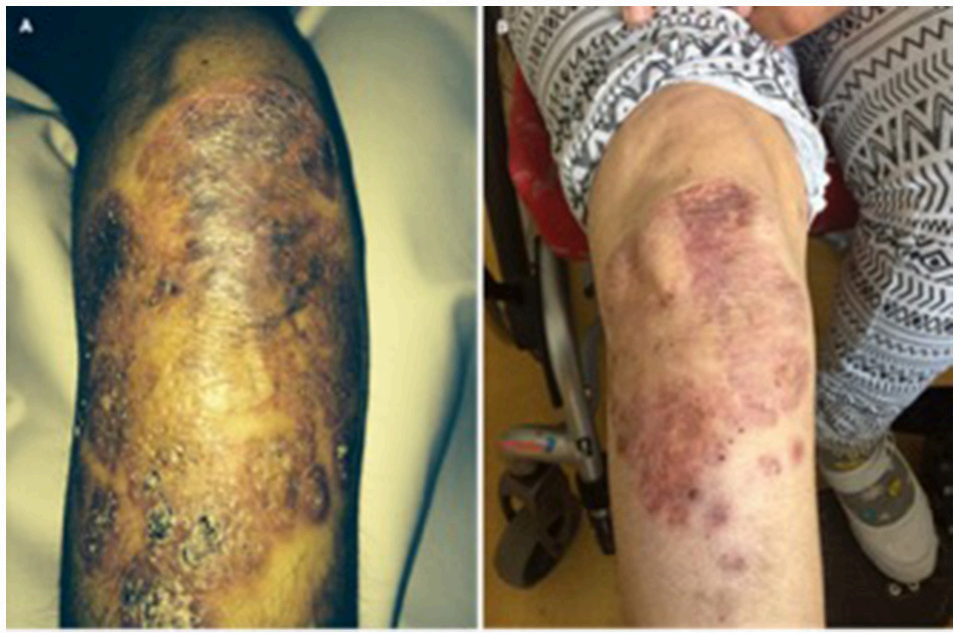
**(A)** Massive skin granuloma in patient 5 and **(B)** regression of granuloma under TNF inhibitor treatment in patient 5 after 1.5 years.

In patient 5, treatment was initiated with adalimumab at a dose of 40 mg every 2 weeks subcutaneously. During treatment, the patient was seen in our outpatient clinic every 6 months. The massive skin manifestation was significantly regressive under the treatment. After 1.5 years, no ulcerations could be detected anymore and the granuloma was significantly reduced in size and extension (Figure 4). Three years after treatment initiation there is an ongoing improvement of the skin granuloma.

In none of the three patients treated with TNF inhibitors side effects were detected. The neurological decline was in accordance with the age and the natural course of their primary disease. No severe or infectious complications were observed during the treatment and follow up period.

In patient 8, who was treated with HSCT, granulomas disappeared completely after reconstitution of his immune system, showing normalized immunoglobulins levels (IgG2 and IgA) and restored T-Cell immunity (). More details on the pre-, per-,and post-HSCT have been described in a separate case report by Bakhtiar et al.^1^. After a 6 year follow up period the patient is still in remission.

### Immunological findings

Immunological features of patients with and without granulomas were compared (Table 2). Interestingly seven of eight patients with granulomas were total IgA deficient, but there were no differences in IgG and IgM levels. All lymphocytes subsets were equally distributed except for naïve CD8 cells which were significantly lower in patients with granulomas.

**Table 2:** Immunological findings.

	**All patients**	**Without granuloma**	**With granuloma**
**Number of patients (n)**	**44**	**36**	**8**
α-feto-protein (ng/mL)	303.4 (36.3–1338)	303.4 (36.3–1338)	289.9 (52.2–603)
IgG (g/L)	8.65 (0.66–20.06)	8.81 (3.66–20.06)	8.36 (0.66–12.77)
IgG_2_ (g/L)	0.68 (0.03–3.74)	0.73 (0.18–3.01)	0.6 (0.03–3.74)
IgM (g/L)	1.21 (0.17–2.50)	1.21 (0.19–2.50)	1.12 (0.17–1.87)
IgA (g/L)	0.05 (0.02–2.00)	0.31 (0.02–2.00)	0.05 (0.04–0.92)^*^
Lymphoycte count (/μl)	1226 (595–2503)	1208 (595–2503)	1209 (910–2289)
CD3^+^ cells (/μl)	700 (282–1943)	703 (282–1907)	643 (432–1943)
CD4^+^ cells (/μl)	363 (101–1589)	389 (101–1389)	311.5 (192–1589)
naïve CD4^+^ cells (/μl)	14.5 (1–360)	16 (2–360)	10.5 (1–311)
CD4^+^ EMRA cells (/μl)	1 (0–14)	1 (0–14)	1 (0–3)
CD4^+^ EM cells (/μl)	97 (37–1093)	89 (37–472)	136 (66–1093)
CD4^+^ CM cells (/μl)	194 (60–1145)	209 (60–1145)	156 (112–363)
CD8^+^ cells (/μl)	212 (36–946)	207 (36–946)	249 (88–316)
naïveCD8^+^ cells (/μl)	26.5 (1–392)	44 (2–360)	17 (5–29)^*^
CD8^+^ EMRA cells (/μl)	21 (0–142)	16 (0–142)	57 (15–120)
CD8^+^ EM cells (/μl)	59 (8–217)	49 (8–217)	83.5 (13–130)
CD8^+^ CM cells (/μl)	56 (13–382)	56 (13–382)	63 (27–158)
Regulatory T cells	22 (1–64)	23 (8–64)	18.5 (1–25)
CD19^+^ cells (/μl)	81 (15–491)	81 (15–345)	93.5 (45–264)
CD56^+^ cells (/μl)	260 (72–894)	279 (71–894)	284 (217–546)

*Statistical analysis using Mann-Whitney-U-Test*.

* *p < 0.05*.

## Discussion

Granulomas without an identifiable infectious agent have been described in various patients with PID, like chronic granulomatous diseases, CVID and A-T (14, 15, 26). It is interesting to note that most descriptions of granulomas have been done in PID with increased cancer risk and defective DNA repair like A-T, NBS and cartilage-hair hypoplasia (14–16, 27).

From our cohort of 44 classical A-T patients, eight patients (18.2%) suffered from progressive granulomas. Histopathology of the lesions confirmed the presence of granulomatous inflammation without detection of any microbiological agent in all eight patients. Moreover, to best of our knowledge we report for the first time that extensive granulomatous inflammation can occur extra-dermal and involve bone, ankle and synovia. Extra-dermal granulomas in various other organs like lung, liver and the gastro-intestinal tract have been described in CVID, in patients with RAG mutations, chronic granulomatous disease (CGD) and other immundeficiencies (28–31). It has been suggested by Chiam et al. (15) that granulomas can be considered as a manifestation of dysregulation in wound healing and tissue repair explained by the immune defects in these PIDs. Next we did a precise analysis of immune function of our cohort of 44 classical A-T patients and compared patients with and without granulomas to find differences in immune competence. Interestingly seven of eight patients with granulomas were total IgA deficient, but there were no differences in IgG, IgG2, and IgM levels. In addition patients with granulomas had significant lower CD8 cells.

Our findings are not in line with a recent paper on cancer risk in A-T patients, IgG2 deficiency, but not total IgA deficiency was found to go along with reduced survival due to cancer even when the analyses were restricted to classical A-T patients (32). Thus, it can be speculated that cancer risk and granulomas do not follow the same impairment of immune regulation. Interestingly all lymphocytes subsets were equally distributed with the exception that patients with granulomas had significantly lower naïve CD8 cells. Neither the cell-biological mechanisms nor the possible clinical implications of low naïve CD8 cells are currently known. Naïve CD8 cells must be activated and undergo differentiation into effector cells, a time-consuming process during which infection may progress. In contrast, CD8 memory cells have a clear advantage over naïve cells in providing protection against infections with viruses or other intracellular microbes (33). The clinical spectrum and immune defect in A-T is variable and related in part to absence or presence of ATM kinase activity (34). A-T variants have a milder clinical phenotype, normal T and B-cell numbers, no granulomas and a longer lifespan compared to classical A-T. In contrast, classical A-T patients suffer from B and T cells deficiencies, a higher cancer risk and have a shorter life expectancy. In addition, most patients with classical A-T show absence of IgA, low IgG2 levels and a restricted B and T cell receptor repertoire pointing to more severe inappropriate immune regulation (35–38).

One limitation of our study was that our tissue sections were not investigated for persisting rubella virus. Several studies hypothesize that persisting rubella virus (RV) vaccine strain is stimulating granuloma formation in PID (31, 39). RV-positive cells in granulomas were identified as M2 macrophages (40). M2 macrophages are crucial for maintaining tissue homeostasis, whereas pro-inflammatory M1 macrophages play an essential role in eliminating pathogens (41). The molecular programs that control the differentiation of such macrophage populations have not been fully elucidated but may involve p53 and TNF signaling to suppress granuloma formation (42). Unfortunately, we were not able to stain our biopsies for RV to elucidate the role of RV in our patients. Since RV vaccination was performed in all A-T patients, persistent RV vaccine strains seems to be more likely an innocent bystander than a causal role in granuloma formation.

The efficacy of TNF inhibitors has been demonstrated in a variety of autoimmune and granulomatous inflammatory diseases such as Crohn's disease, sarcoidosis, and rheumatic diseases (43–45). In addition, there are several optimistic case reports that treatment with infliximab is effective in patients with granulomas and an underlying PID (22, 23, 46). This prompted us to treat three of our most affected A-T patients with TNF inhibitors, although there is concern about its use in patients with PIDs (16). Treatment success with TNF inhibitors was variable as reported previously (22, 23, 27, 47). In one patient with granulomatous inflammation within the knee synovia, treatment led to a total remission. In two patients, treatment with TNF inhibitors led to a partial regression of granulomas. Interruption of treatment in one patient caused deterioration again and he is now continuously treated for 7 years without any side effects. In the group of TNF inhibitors, only infliximab is administered intravenously. Infusion reactions are often presented as hypersensitivity reactions. Acute cutaneous reactions, such as urticaria or erythematous rashes, occur within 2 h of the onset of infusion and affect approximately 5–23% of patients and severe reactions are rare (46). However, in our patients all infusions were well tolerated. Therefore we recommend a trial of TNF inhibitors in all patients with large skin and extra-dermal granulomas. According to our experience a clinical treatment success should be visible after 6 months.

It is important to note that granulomas in A-T underlie a slow progression over years in all untreated patients and can lead to significant morbidity. Treatment with TNF inhibitors was safe and partially successful in our patients. Although treatment with TNF inhibitors is effective, restoring the immune function by HSCT is the best strategy to overcome the inappropriate immune regulation in PID. This is demonstrated by patient 8. HSCT lead to complete remission of granulomas at the proximal interphalangeal joint of his middle finger and small skin lesions at his elbow. This corresponds with the favorable results of BMT in patients with cartilage-hair hypoplasia, demonstrating again that HSCT presents the only curative treatment (27). In the future HSCT is a promising approach offering a new avenue for therapeutic options for A-T, especially if performed early, during stages of limited disability.

In conclusion granulomas in A-T progress slowly over years and can lead to significant morbidity. Treatment with TNF inhibitors was safe and in part successful in our patients. Interestingly HSCT leads to complete remission, and indicates that aberrant immune function is responsible for granulomas in A-T patients.

## Ethics statement

This study was carried out in accordance with the recommendations of the Declaration of Helsinki and consistent with Good Clinical Practice and the applicable regulatory requirements. The protocol was approved by the responsible ethics committees in Frankfurt. All subjects gave written informed consent in accordance with the Declaration of Helsinki.

## Author contributions

SW contributed to the conception and design, participated in the data acquisition, contributed to the analysis and interpretation of the data and drafted the article. EV contributed to the conception and design, participated in the acquisition of data and critically revised the article. SB, HP, and LP participated in the data acquisition and critically revised the article. RS contributed to the data analysis and interpretation and critically revised the article. SZ contributed to the conception and design, participated in the data acquisition, contributed to the analysis and interpretation of the data and critical revision of the article.

### Conflict of interest statement

The authors declare that the research was conducted in the absence of any commercial or financial relationships that could be construed as a potential conflict of interest.

## Footnotes

^1^Bakhtiar A, Woelke S, Huenecke S, Taylor M, Kieslich M, Schubert R, et al. Pre-emptive allogeneic hematopoietic stem cell transplantation in ataxia telangiectasia. Case Report. *Front. Immunol*. (under review) 27.

## Supplementary material

Figure S1 Slow progression of granulomas in patient at the knee **(A)** after 5 years and **(B)** after 6 years.

Figure S2 Multiple skin granulomas in patient 2 on the **(A)** forearm and **(B,C)** lower leg.

Figure S3 Multiple skin granulomas in patient 3 on the **(A)** arm and **(B)** lower leg.

Figure S4 Joint granuloma in patient 8. **(A)** Swelling of the finger joint and **(B)** X-rays of the finger joint before BMT.

## Abbreviations

A-T: Ataxia telangiectasia
ATM: Ataxia telangiectasia mutated
BMT: bone marrow transplantation
CVID: Combined variable immunodeficiency
CGD: Chronic granulomatous disease
CM: Central Memory
EDTA: Ethylenediaminetetraacetic acid
EM: Effector Memory
EMRA: Effector Memory RA^+^
NBS: Nijmegen Breakage syndrome
PID: Primary Immunodeficiency
PI3 kinase: phosphatidolinositol-3 kinase
RV: rubella virus
TNF: Tumor necrosis factor.
