# Supplementary figures and images for: Treatment of Granulomas in Patients With Ataxia Telangiectasia
Source: Front Immunol. 2018 Sep 18;9:2000. doi: 10.3389/fimmu.2018.02000 (PMC6153364; doi:10.3389/fimmu.2018.02000)

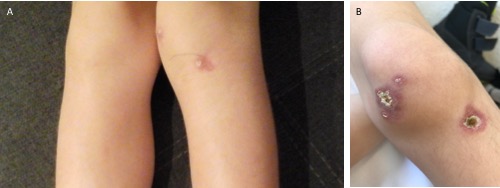

Supplement: Figure S1 — Slow progression of granulomas in patient at the knee (A) after 5 years and (B) after 6 years. [file Image_1.JPEG]

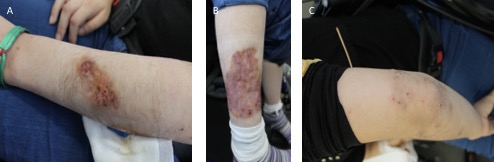

Supplement: Figure S2 — Multiple skin granulomas in patient 2 on the (A) forearm and (B,C) lower leg. [file Image_2.JPEG]

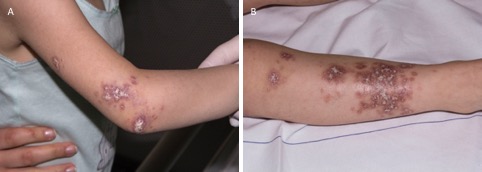

Supplement: Figure S3 — Multiple skin granulomas in patient 3 on the (A) arm and (B) lower leg. [file Image_3.JPEG]

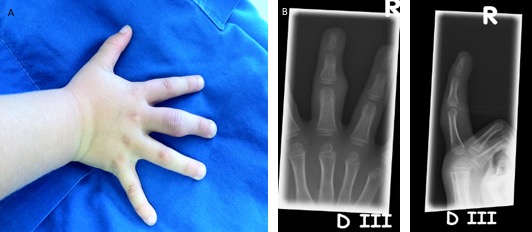

Supplement: Figure S4 — Joint granuloma in patient 8. (A) Swelling of the finger joint and (B) X-rays of the finger joint before BMT. [file Image_4.JPEG]
